# Activation and execution of lipoxygenase catalysis

**DOI:** 10.1016/j.rbc.2026.100076

**Published:** 2026-03-24

**Authors:** Alan R. Brash, Ernst H. Oliw

**Affiliations:** a Department of Pharmacology and the Vanderbilt Institute of Chemical Biology, Vanderbilt University, Nashville, TN, USA; b Department of Pharmaceutical Biosciences, Uppsala University, Uppsala, Sweden

**Keywords:** Lipoxygenase, Mechanism, Molecular oxygen, Dioxygenase, Linoleic acid, Arachidonic acid, Eicosanoids, Oxylipins

## Abstract

As noted in Gordon A. Hamilton’s classic review “Chemical Models and Mechanisms for Oxygenases”, the direct reaction of a triplet molecule (i.e. O_2_) with a singlet to give singlet products is a spin-forbidden process (*in* Molecular Mechanisms of Oxygen Activation, O. Hayaishi (Ed), Academic Press, NY, pp. 405–451, 1974). As lipoxygenases are among the enzymes that do not activate molecular oxygen, activation of the fatty acid substrate is required. Furthermore, lipoxygenases do not bind the reacting O_2_ molecule and it is free to travel wherever diffusion takes it. On top of all this, lipoxygenase enzymes are in a catalytically inactive state at rest. Within this set of limitations, here we consider lipoxygenase activation and the challenges overcome in execution of catalysis including activation of the catalytic metal, issues of substrate orientation, O_2_ channels, hydrogen abstraction, and residues that affect regio- and stereo-specificity of the product hydroperoxides.

The Hamilton review quoted in the Abstract (and available currently via Google Books) goes on to explain that molecular oxygen O_2_ is a diradical with two unpaired electrons, meaning it exists as ·O–O· (rather than O=O) and in this triplet state allowing only two ways for its reaction with organic molecules (singlets, with all electrons paired) – either via O_2_ coupling to a transition metal (not relevant for lipoxygenase biochemistry) or O_2_ reacts with a substrate free radical created by the enzyme – the basis of fatty acid oxygenation in LOX catalysis [1]. Although beyond the scope of this review, we like Borden et al. for a readable Abstract and Introduction explaining why diradical O_2_ molecules do not self-polymerize forming oligomers, O_2_–O_2_–O_2_–O_2_ etc., as might be expected from the usual favorable coupling of radical species; in short, strong intramolecular resonance stabilizes the unpaired electrons in triplet O_2_ allowing its accumulation as one of the most abundant molecules in Nature [2].

The LOX enzyme creates the substrate free radical by hydrogen abstraction from the polyunsaturated fatty acid and O_2_ then reacts readily with the now activated fatty acid. The basis of this substrate activation and control of the reaction with O_2_ is what individual LOX enzymes are designed to accomplish, and we begin here with a look at the key enzyme catalytic center in the LOX enzyme family, with remarkable parallels in the active site to superoxide dismutases (SOD).

## 1. The Catalytic Center of Lipoxygenase Enzymes

### Overall structures of LOX enzymes -

**Fig. 1.**
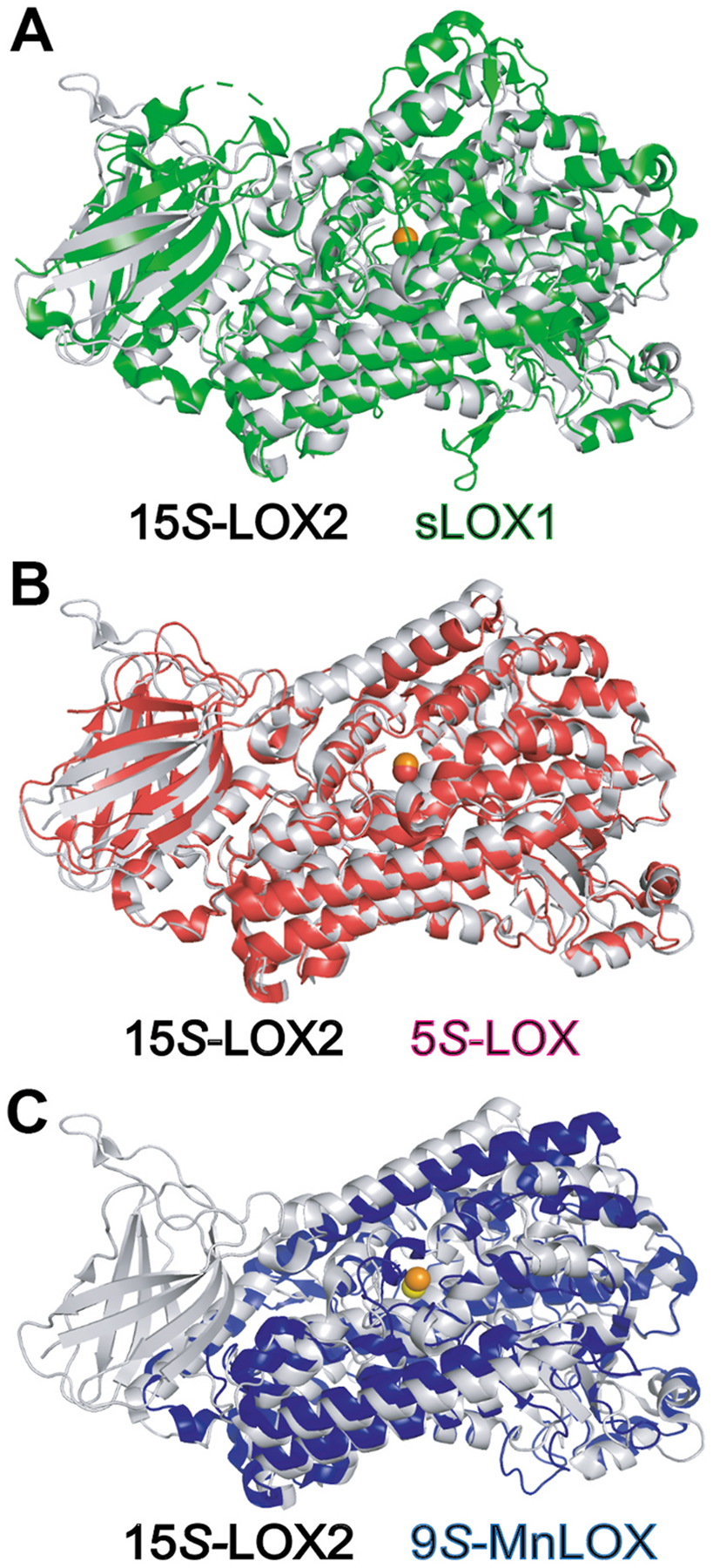
Structural comparison of human, plant and a fungal MnLOX A: Superposition in PyMOL of 15*S*-LOX2 (grey) with soybean LOX-1 (green), Fe atom in orange, C_α_ atoms with a root-mean square deviation (RMSD) 1.73 Å. B: 15*S*-LOX2 (grey) and 5-LOX (red), RMSD 1.13 Å. C: 15*S*-LOX2 (grey; Fe orange) and fungal 9*S*-MnLOX (blue; Mn yellow), RMSD 2.82 Å, indicating the MnLOX α-helices align poorly although retaining the general protein fold. Fungal LOX lack the beta-barrel of animal and plant LOX. The C-terminal ends of each structure are visible at the upper left of the catalytic metals. 15*S*-LOX2, 5-LOX and 9*S*-MnLOX structures were from the Protein Data Bank and 12*R*-LOX from the Alphafold Protein Structure Database.

**Fig. 2.**
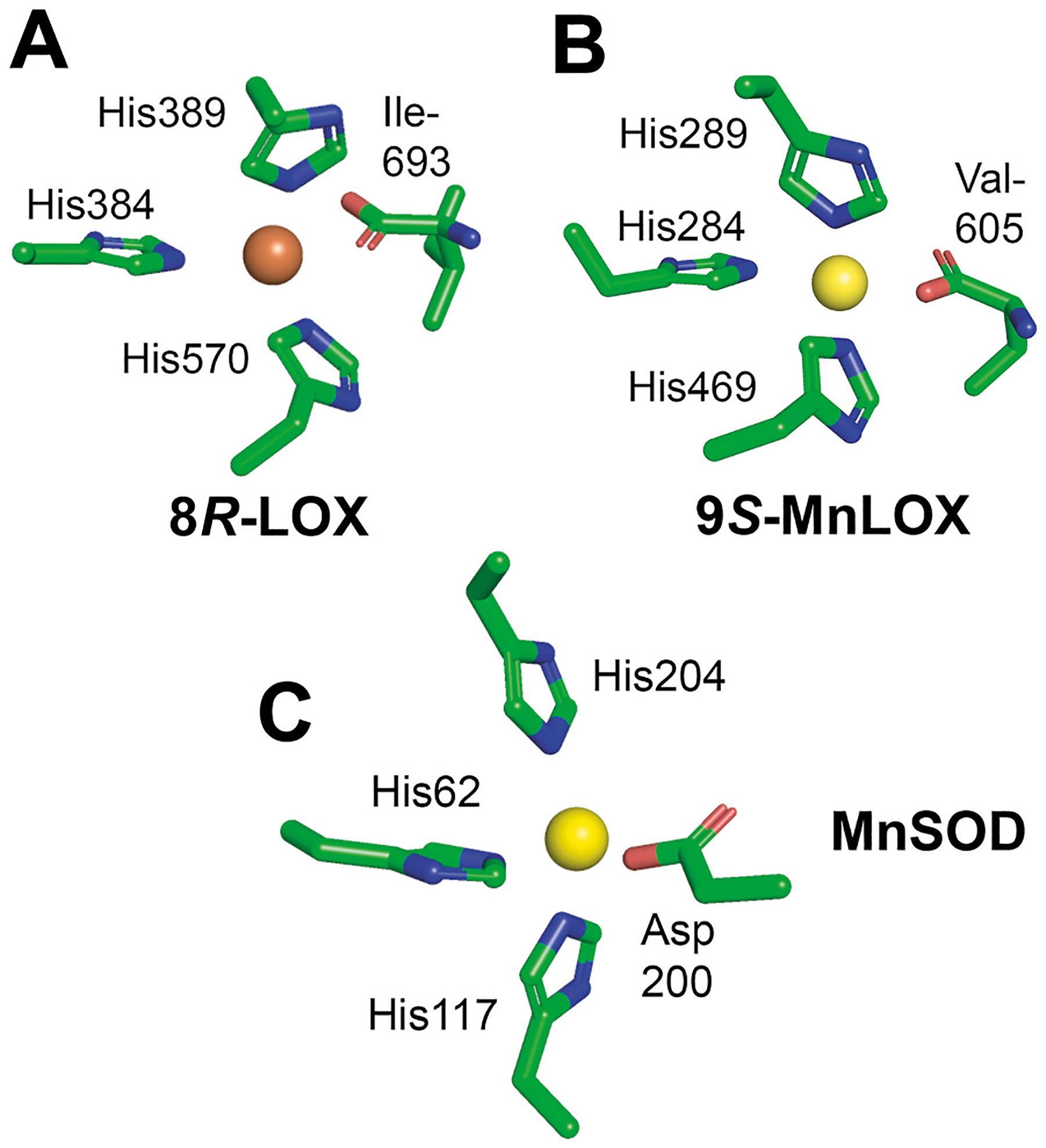
The four metal ligands of FeLOX, MnLOX and SOD A. The metal ligands of 8*R*-LOX (PDB 4QWT) are essentially conserved in all FeLOX; Fe in orange. B. 9*S*-MnLOX (PDB 5FNO), Mn in yellow. C. The four metal ligands of MnSOD of a cyanobacterium (PDB 1gv3; *Nostoc* sp.), which are conserved in this family of Fe-, Mn-, and Fe/MnSOD; Mn in yellow.

The structures set the stage for analysis of LOX activation and execution, Fig. 1 (e.g. Refs. [3–11]). All share the catalytic domain with non-heme ferrous iron (e.g. refs [12–14], [15]) with the exception of LOX of certain fungi, which contain manganese [16–20]. Common to all LOX are the metal ligands, with three fixed histidines, plus a His, Asn or Ser, and significantly, the terminal carboxyl of the polypeptide chain, usually on Ile, very occasionally Val (see later, Fig. 2), – and therefore no C-terminal His tags on recombinant LOX enzymes! The beta-barrel or C-domain is common to plant and animal LOX with some of the microbiological enzymes lacking this lipid-associating N-terminus. Four critical helices of the catalytic domain make the substrate entry and binding channel, and along with oxygen access, the subject of many computational analyses, are considered later in the review. Some of the structures lack an open substrate access route, adding to the challenge of modeling computations which not infrequently predict an inappropriate substrate orientation for soybean lipoxygenase [21–23].

The LOX reaction RH + O_2_ → ROOH is a balanced equation, no cofactor is required, and hence mixing substrate and enzyme leads to product formation, a fact that confounded attempts to crystallize a LOX enzyme-substrate complex. Finally, this was achieved for 8*R*-LOX with bound arachidonic acid under anaerobic conditions [24], providing insights that helped with informed modeling in other LOX enzymes (e.g. Ref. [25]). Advent of the 8*R*-LOX-AA structure confirmed the predicted “tail-first” substrate binding orientation and highlighted the role of multiple amino acid residues in arranging the position of AA for catalysis [24], all points considered later in this review.

### The catalytic metal, Fe or Mn -

In 1974 Pistorius and Axelrod reported Fe in soybean lipoxygenase (sLOX) and initiated studies of the catalytic metal using EPR [12,26]. These early analyses showed that native sLOX lacks EPR signals, but treatment with 13*S*-HPODE induces signals of high spin Fe^3+^ [26,27]. It is now generally acknowledged that lipoxygenases cycle between the inactive Fe^2+^ enzyme with 6 d-electrons (EPR silent) and the high-spin active Fe^3+^ forms with 5 d-electrons [28,29].

The crystal structure of sLOX shows that in the “first coordinating sphere” Fe is ligated to nitrogen of three His residues and the oxygen atom of the C-terminal carboxylate group [3,4,30]. This 3-His-1-carboxylate motif is conserved in plant and animal lipoxygenases and the carboxylate is usually provided by Ile (Fig. 2A) [7,31]. A solvent molecule is also ligated to the iron and the second coordinating sphere (close but not directly liganded) usually contains an Asn or His residue.

Fungi express LOX with Mn as the catalytic metal and the crystal structures reveal a similar 3-His-1-carboxylate motif with the carboxylate of Ile replaced by a C-terminal Val residue, Fig. 2B [32,33] Mn and Fe are sequential neighbors in the periodic table and the ionic radii of Mn and Fe ions are almost identical. It is therefore not surprising that Mn- and FeLOX both can bind the other metal but this occurs with loss of catalytic activities [23,34,35]. This is likely due to the different reduction potentials of Mn and Fe (near 1.5 and 0.77 V, respectively). Redox potential determines the relative ability to lose or gain electrons as is prerequisite for oxidation-reduction cycling in catalysis. For free Mn the high positive value enhances its ability to acquire electrons (and be reduced), with adverse consequences for its oxidation. For the steps involving oxidation of the metal both Fe- and MnLOX proteins must reduce the redox potentials of the metals but MnLOX needs to be reduced more substantially.

### Fe-, Mn-, and Fe/Mn-superoxide dismutases -

There are insights on this regulation of activity with catalytic Fe or Mn from superoxide dismutases (SOD), as the family encompasses enzymes using either metal (designated Fe/MnSOD) [36]. Their metal ligands are also three His residues, the carboxyl group of an Asn residue and a coordinated solvent molecule (Fig. 2C) and Trp and Gln residues in the second coordinating sphere (not shown). LOX enzymes and these SOD use the same metal ligands although the exact geometry of the amino acids differs (Fig. 2). Mutational studies of the Fe/MnSOD subfamily illustrate that the second coordinating sphere of amino acids can adjust the redox potentials of both metals to levels of catalytic competence.

The concepts and principles are outlined exceptionally well in A.F Miller’s review on “Superoxide dismutatses: Ancient enzymes and new concepts” [36]. Readers will also appreciate the 13-verse poem, yes poem, that opens Miller’s paper on proton-coupled electron transfer (PCET) in Fe- and MnSOD, “How doth the protein big and slow - control whence electrons come and go?… ….” and continuing with technically correct mechanism! [37]. To be functional with either metal, Fe/MnSOD enzymes are predicted to apply redox tuning intermediate between the tuning applied by the metal-specific SOD. It is concluded that the evolution of the metal-specific enzymes likely occurred via Fe/MnSOD intermediates and without “interruption of service” [36]. For LOX enzymes, evolution of Fe- and MnLOX may also have occurred via an ancient fungal Fe/MnLOX although the latter is not as yet identified. For the separate families of Fe- and MnLOX enzymes, adjustments to the redox potential are imposed by neighboring residues and the environments are optimized for MnLOX and FeLOX individually, and are not appropriate when the metals are switched.

## 2. Structure of Lipoxygenase Substrates

### Sites for fatty acid oxygenation -

**Fig. 3.**
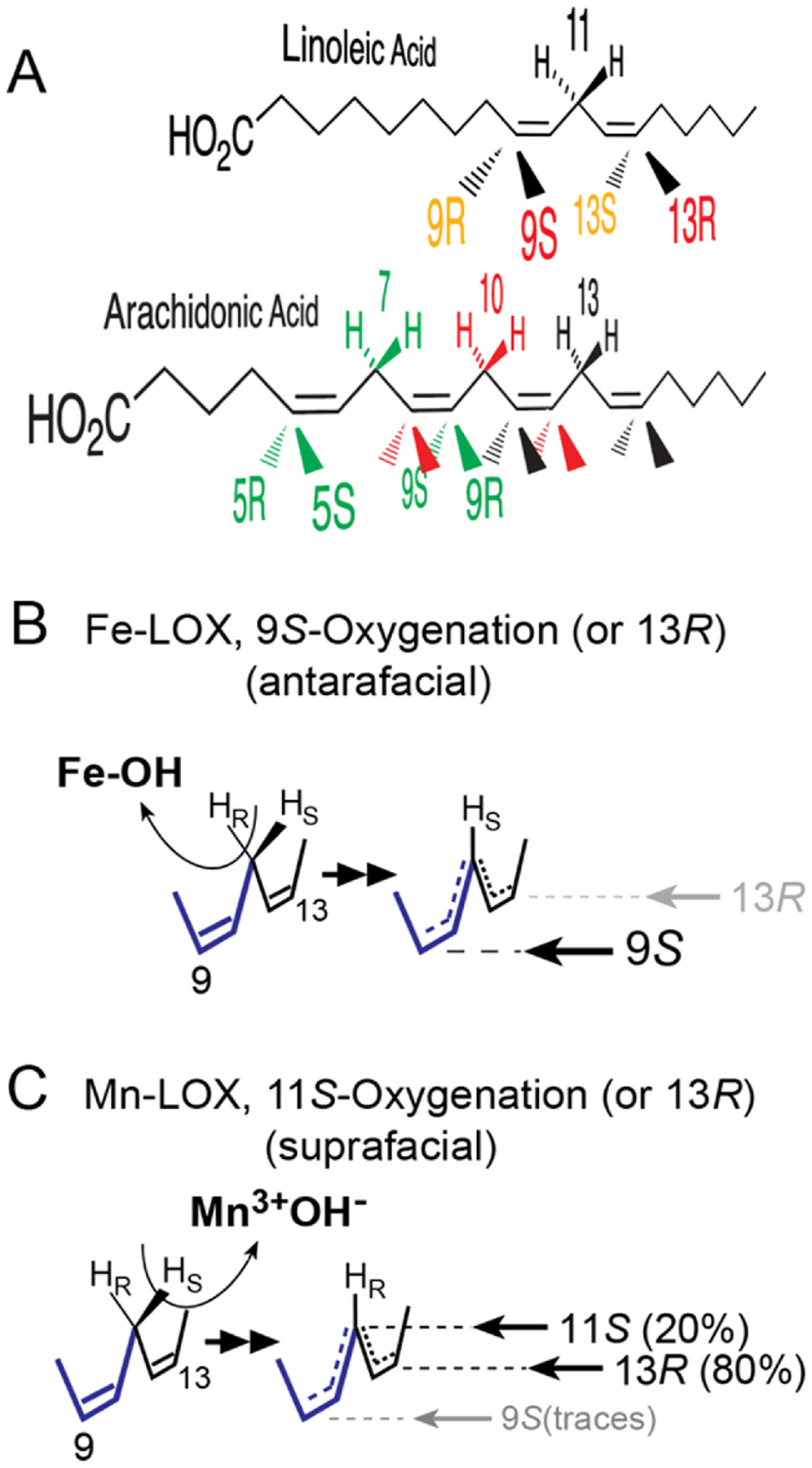
Available positions for oxygenation in linoleic and arachidonic acids and H-abstraction by Fe- and MnLOX **A**: Perspective view of linoleic and arachidonic acids with colors associated with each pentadiene moiety. There are three reactive pentadienes on arachidonic acid, and for clarity not all the positions are labeled. B: FeLOX catalyze antarafacial hydrogen abstraction and oxygenation, in this case abstraction of the 11*pro-R* hydrogen and formation of 9*S*- or 13*R*-hydroperoxides of linoleic acid. C: MnLOX catalyze suprafacial H-abstraction and oxygenation, in this case removal of the 11*pro-S* hydrogen and formation of 11*S*- and 13*R*-linoleate hydroperoxides.

Hydrogen abstraction from the CH_2_ between the two double bonds of linoleic acid creates four reactive sites at the ends of the activated pentadiene (9*R*, 9*S*, 13*R*, and 13*S*), and up to twelve on arachidonic acid, all which are reported individually as LOX products, Fig. 3A. In the Fe-LOX the stereospecific H-abstraction and oxygenation are antarafacial (on opposite sides of the reacting pentadiene), a point established in over a dozen different LOX reactions [38], and illustrated in Fig. 3B on the pentadiene of linoleic acid in catalysis of 9*S* or 13*R* oxygenation. The linoleate Mn-LOX differ in two ways. They catalyze suprafacial hydrogen abstraction and oxygenation (on the same side of the C9–C13 pentadiene) and they can insert O_2_ not only at the ends of the pentadiene, but also in the middle position (C11), Fig. 3C. Furthermore, MnLOX isomerize the hydroperoxide at C11 to hydroperoxides at C9 or C13 as end products. Oxidation of the bis-allylic CH_2_ at C11 can also be catalyzed by a cyanobacterial Fe-LOX, although this enzyme does not isomerize the hydroperoxide to other positions [39].

## 3. Activation of the Lipoxygenase Enzyme

### First things first: activation of the lipoxygenase -

Native Fe-LOX enzymes are inherently inactive. They have the catalytic iron in the ferrous state and incapable of activity. With the long-standing availability of pure soybean LOX-1 as a model, it has been recognized for around 50 years that activation requires oxidation to the ferric iron and that this is achieved by interaction with fatty acid hydroperoxide product [27,40].


$${\text{Fe}}^{2+}+\text{R}–\text{O}:\text{OH}\to {\text{Fe}}^{3+}-\text{OH}+\text{RO}\cdot$$

Starting with pure fatty acid substrate, this transformation of ferrous to ferric can take seconds or minutes as product slowly becomes available, and is evidenced by a lag phase in catalysis until the enzyme achieves full activation. The enzyme can be activated immediately and the lag phase all but eliminated by addition of a few equivalents of hydroperoxide product (e.g. Refs. [41–46]). With dilute enzyme, traces of oxidants in solution can lead to activation [44].

The oxidation of Mn^2+^ requires more energy than the oxidation of Fe^2+^ to Fe^3+^. Accordingly, the MnLOX have prolonged lag phases, although they are also activated by small amounts of hydroperoxides. Mn-substituted FeLOX lack catalytic activities, which is surprising as the metal ligands are identical and appear to bind the catalytic metals in the same way. The redox potentials for M^2+^ → M^3+^ + e^−^ of the two metals differ by a factor of two, and apparently this is a hurdle not overcome in Mn-substituted FeLOX.

### Activation requires O_2_ –

For activation, a fly in the ointment arises from experimental observations showing that molecular oxygen, O_2_, is involved in the hydroperoxide oxidation of ferrous to ferric LOX. On the face of it, having the HPETE or HPODE oxidize the iron should do the job, so where does O_2_ have a role? A good part of the evidence comes from two LOX reactions that exhibit a particularly prolonged lag phase of activation. And to be considered later are questions on the relevance of a classic study of HPODE transformations associated with the visible color changes associated with mixing milligrams of pure LOX enzyme with fatty acid hydroperoxide.

To the best of our knowledge Ivanov and coworkers were the first to implicate O_2_ in activation to the ferric enzyme [47]. They analyzed oxygenation of 19-HETE by reticulocyte 15-LOX, a sluggish reaction with prolonged lag phase, and the hydroperoxide-dependent activation of ferrous to ferric (exemplified by the long lag phase) was strongly influenced by the O_2_ concentration [47]. The authors observed that the activating HPODE was converted to oxo-dienes (fatty acid ketones and aldehydes with smooth-looking UV chromophores with lambda-max around 280 nm), although the connection of this to the activation process was not directly discussed. We note here that oxo-dienes are the major products of the anaerobic reactions of conventional lipoxygenases with fatty acid substrates and their products [48], with the potential implication that the enzyme is responding to a lack of active site O_2_, a point developed in the next reaction considered.

### Mechanistic basis of the prolonged lag phase with eLOX3 –

**Fig. 4.**
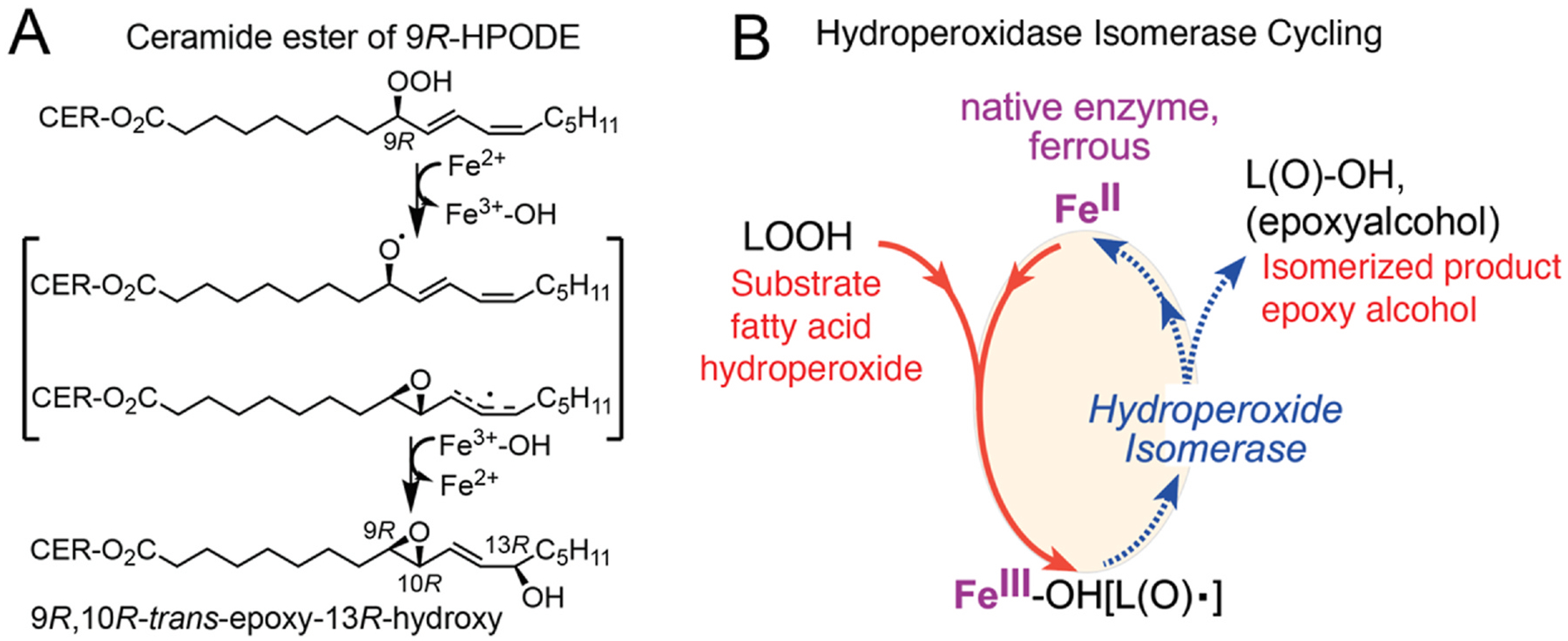
eLOX3 acts as a hydroperoxide isomerase **A**: In mammalian epidermis eLOX3 isomerizes 9*R*-HPODE ceramide ester to an epoxyalcohol (9*R*,10*R*-*trans*-epoxy-13*R*-hydroxy); B: Hydroperoxidase cycling. HPODE substrate (top left) oxidizes the LOX ferrous iron to ferric, forming a Fe–OH^III^(LO^•^) complex (bottom structure) that undergoes isomerase cycling to epoxy-alcohol with return of the iron to ferrous [49,50]. Figure B is adapted from ref [54].

A second LOX system with an especially long lag phase for activation involves one of the mammalian LOX enzymes, epidermal lipoxygenase-3, eLOX3. In fact, under conventional conditions of incubation eLOX3 has no demonstrable reaction with natural polyunsaturated fatty acid substrate [49–51]. It will exhibit dioxygenase activity under certain experimental conditions, albeit with a long and O_2_-dependent lag phase [51]. Despite this deficit in conventional dioxygenase activity, eLOX3 is identified as a causative gene of ichthyosis in human families with inactivating mutations [52], and its mouse knockout shares the lethal phenotype of other genes with an essential role in forming the skin permeability barrier [53]. Its normal or physiological role is in the transformation of linoleate 9*R*-hydroperoxide esterified to omega-hydroxy-ceramide and produced by its upstream partner enzyme, in the skin the partner being 12*R*-LOX [50,52,54]. In this capacity, eLOX3 functions as a fatty acid hydroperoxide isomerase [50]. It converts the linoleate product of 12*R*-LOX, 9*R*-hydroperoxy-octadecadienoate, to the epoxy alcohol 9*R*,10*R*-*trans*--epoxy-11*E*-13*R*-hydroxy-octadecenoate [54], Fig. 4A.

This hydroperoxide isomerase cycling reaction by eLOX3 involves the same first step as in conventional activation of a LOX enzyme from ferrous to ferric, Fig. 4B; the added fatty acid hydroperoxide group is cleaved homolytically, (conceptually RO:OH to RO· and ·OH), with RO· being an alkoxyl radical and the ·OH species oxidizing the ferrous iron to ferric as in conventional LOX oxygenations. In executing this first step in the cycle, the enzyme activation complex exists transiently with bound fatty acid alkoxyl radical, Fe^3+^-OH[LO•] (bottom of the cycle). Hydroperoxide isomerase cycling is completed by the Fe^3+^-OH[LO^•^] intermediate dissociating to product epoxy-alcohol with return to the initial ferrous state of the iron, Fig. 4B.

### Linking hydroperoxide isomerase and conventional LOX activity -

Key to understanding the connection between this hydroperoxide isomerase activity of eLOX3 and conventional LOX oxygenation is to consider why, in eLOX3, does the ferric enzyme produced by interaction with fatty acid hydroperoxide not go on to catalyze normal LOX activation? According to one proposal, at least part of the answer lies in the access of molecular oxygen to the enzyme active site during activation [55]. Part of this proposal contends that hydroperoxide isomerase cycle will only occur in the *absence* of intervention by O_2_. Molecular oxygen reacts with free radicals instantaneously and with zero activation energy, so its absence from this hydroperoxide isomerase cycling is paramount. Therein stands the difference with normal LOX catalysis.

**Fig. 5.**
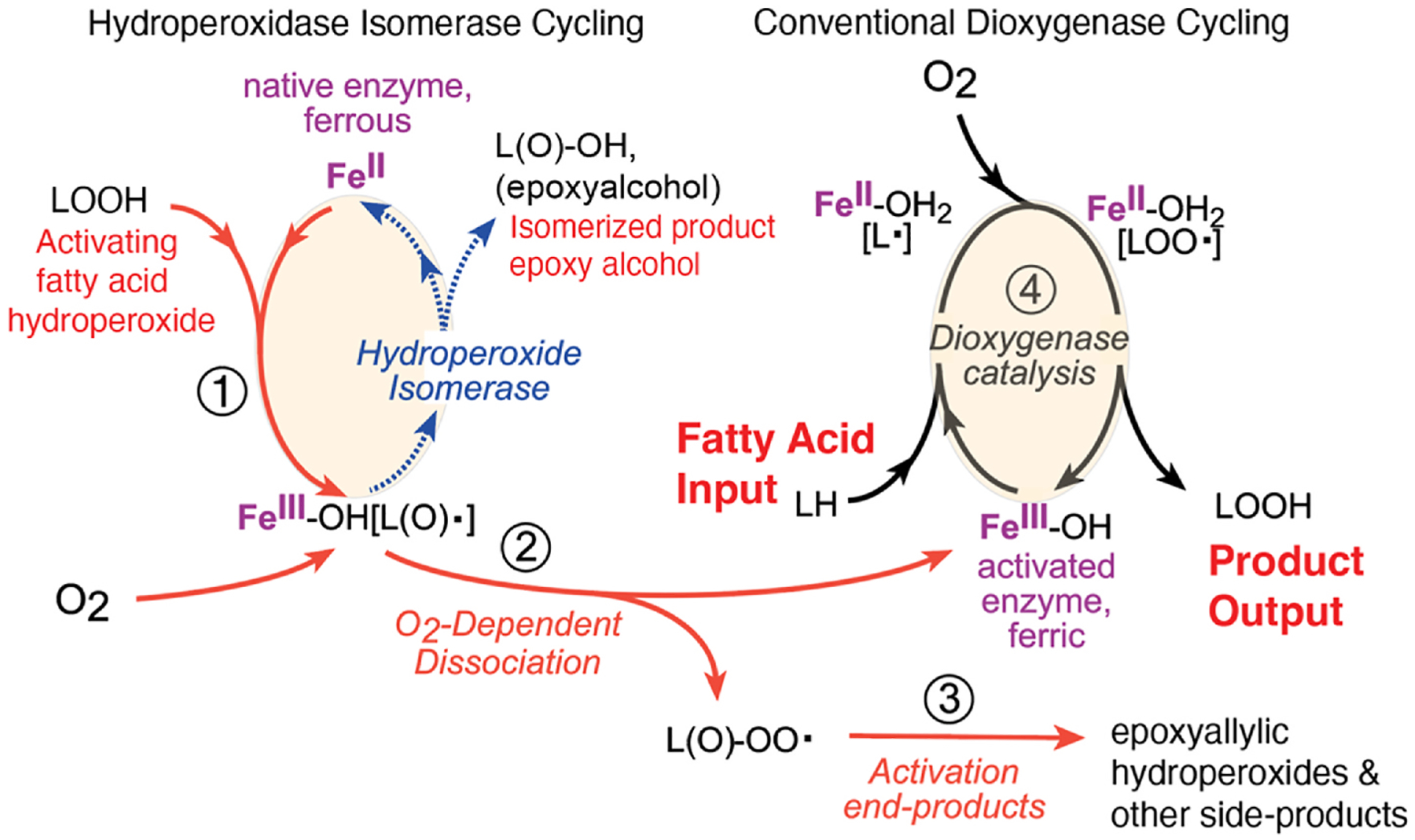
Hydroperoxidase cycling as an activator of conventional LOX-catalyzed oxygenation Fatty acid hydroperoxide (LOOH, left hand side) oxidizes the metal center from ferrous to ferric (or Mn^2+^ to Mn^3+^), and unlike in hydroperoxidase cycling the Fe–OH^III^(LO^•^) complex dissociates in an O_2_-dependent manner to release the Fe^3+^ enzyme for oxygenation reactions (right-hand cycling). Conventional LOX oxygenations require only a single activation event for multiple rounds of fatty acid hydroperoxide production. The figure is adapted from Ref. [55].

**Scheme 1.**
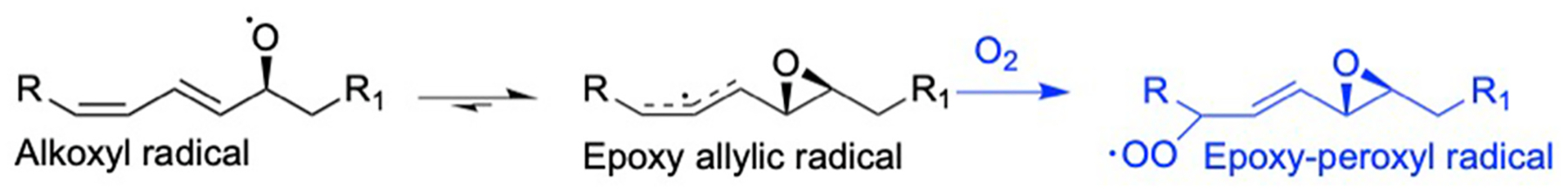
Polyunsaturated fatty acid alkoxyl radicals mainly exist as carbon-centered epoxy-allylic radicals and react with O_2_ to produce peroxyl radicals.

When O_2_ has access to these transformations, the outcome after formation of the Fe^3+^-OH[LO^•^] complex is changed from hydroperoxide isomerase to conventional dioxygenase activity. With access available, molecular oxygen will react with the enzyme-bound alkoxyl radical, and the next part of the proposal contends this is *a required step in clearance of the active site of the oxidation products*, leaving the ferric iron free for conventional dioxygenase cycling, Fig. 5. Of practical significance too, this reaction will also clear the active site of the molecule of O_2_ (as the fatty acid peroxy radical). To follow the experimental results in support of this hypothesis, here it is helpful to recognize that polyunsaturated fatty acid alkoxyl radicals are in equilibrium with, and mainly exist as, carbon-centered epoxy-allylic radicals [56]; and their reaction with O_2_ will produce an epoxy-peroxyl radical, Scheme 1.

In analysis of the fate of the activating fatty acid hydroperoxide in soybean LOX-1 catalysis, products of the activating HPODE were identified as isomeric 9-hydroperoxy-12,13-epoxides [55]. It is proposed these are derived from the oxygenated species (epoxy-peroxyl radical) released from the active site, allowing access by a substrate molecule with ensuing normal LOX oxygenation. In summary, the proposal is that step-1 in Fig. 5 is activation of the ferrous iron to a ferric species with bound fatty acid radical, step-2 is oxygenation of the enzyme bound radical, facilitating its release from the active site, and step 3 is capturing of a hydrogen or other fates to produce side products of LOX enzyme activation. These conjectures appear to “make sense” and fit with the experimental observations although currently there is no direct evidence on the relative affinity of enzyme-bound fatty acid radicals in its support.

### A classic study on LOX-HPODE interaction and potential limitations -

**Scheme 2.**
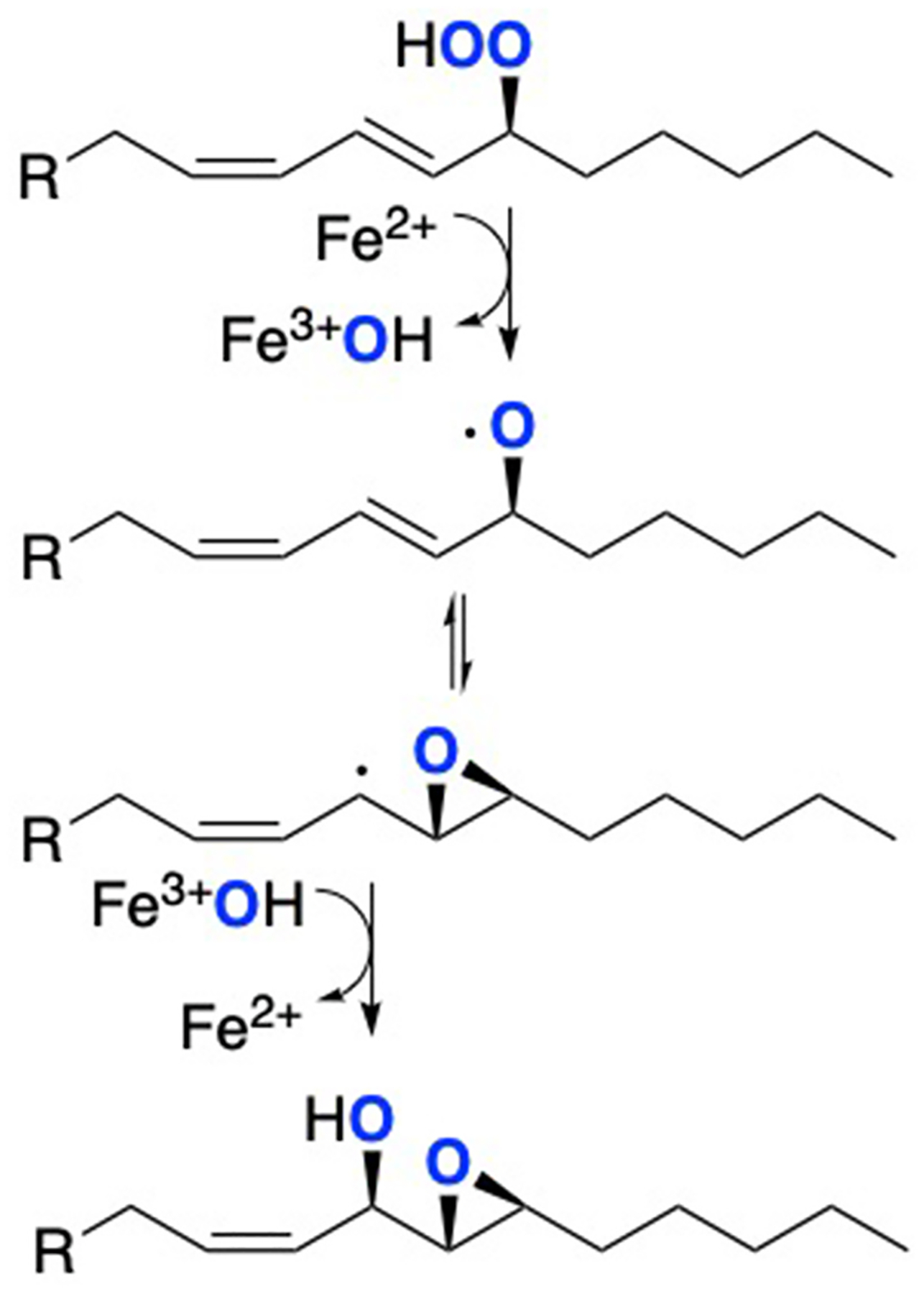
Intramolecular retention of hydroperoxy oxygens in LOX-catalyzed isomerization of 13*S*-HPODE to 11*R*-hydroxy-12*S*,13*S*-*trans*-epoxide.

It is of interest and potential mechanistic relevance that the finding of 9-hydroperoxy-12,13-epoxides upon mixing HPODE activator with soybean LOX-1 [55] conflicts with the results of an oft-quoted classic study of LOX-1/HPODE interaction [57]. In retrospect, this classic study successfully and correctly identified the question addressed, namely the products formed during HPODE-induced color changes to milligrams of enzyme.^1^ One issue is its relevance to normal LOX enzymology, for as we point out here, the high HPODE concentration out-matched the available O_2_ by up to 50-fold. The major product was identified as a *threo*-11-hydroxy-12,13-*trans*-epoxide (Scheme 2). It was formed from 13 μmoles (4 mg) of 13-HPODE in 1.6 ml, greatly exceeding the available O_2_ (~0.25 μmol/ml), which even if replenished to some extent over an hour at room temperature, was leaving the enzyme anaerobic. This does not mirror the normal conditions of LOX catalysis and the study bears further analysis with “physiological” levels of LOX enzymes to further define the products associated with activation (cf. [47,55,58]). For a more recent conundrum of anaerobic LOX reaction, consider the formation of HETEs (not hydroperoxides), formed by hydroxylation with ^18^O from ${\text{H}}_{2}^{18}\text{O}$, in the anaerobic reaction of the A451G mutant of eLOX3 in the presence of 13*S*-HPODE [55].

## 4. Substrate Entry and Binding - Orientation Issues

### Binding of fatty acid substrate: tail-first and head-first orientation -

**Fig. 6.**
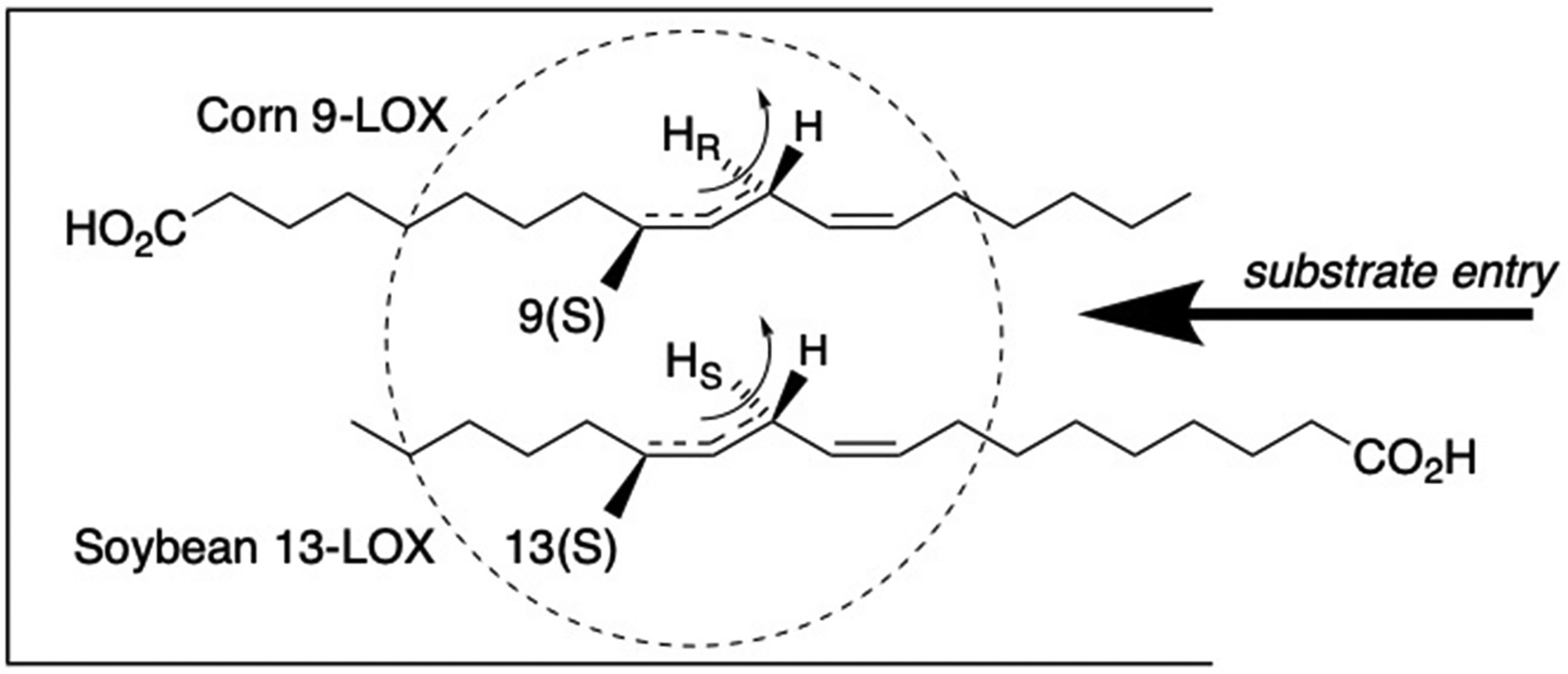
Reverse orientation of substrate in association with opposite hydrogen abstractions in formation of 9*S* or 13*S* linoleate hydroperoxides [60]. “Tail-first” entry in soybean LOX and 11*S* hydrogen abstraction gives 13*S*-HPODE product, carboxyl end entry into corn 9-LOX and 11*R* hydrogen abstraction gives 9*S*-HPODE.

Before there was any knowledge of LOX enzyme structure, there was strong evidence implicating different head-to-tail orientations of fatty acid binding during catalysis. In 1972, Egmond and coworkers reported on the metabolism by soybean and corn (maize) LOX of linoleic acids stereospecifically labeled with tritium on the 11-carbon [59]. The results showed that, whereas soybean LOX abstracted the 11-pro-*S* hydrogen in the synthesis of 13*S*-HPODE, the corn LOX removed the 11-pro-*R* hydrogen in forming 9*S*-HPODE, Fig. 6.

The authors were perceptive in concluding, quote: “These results indicate that if the active sites of both enzymes are stereochemically identical, the differences in positional and stereochemical configuration of the hydroperoxides result from an alternate orientation of the substrate molecule on the enzymes” [59]. Perceptive in 1972 and correct to this day.

### Identifying the orientation in the LOX enzyme -

As to which orientation was which in the above experiments, assignment begins with the assumption of a single access channel into the active site. (A single entrance channel is likely for the great majority of LOX enzymes, although for some there may be a “back door” entrance, see later, a U-shaped channel open at both ends). With only one way in, the fatty acid could reach the active site by entering “tail-first” or “head-first” (carboxyl end first). Again, prior to any knowledge of enzyme structure, it was established beyond doubt that for 15-LOX enzymes and selected others the substrates enter tail-first. The evidence rests securely on analysis of the metabolism of large esters of the polyunsaturated fatty acid substrate; 15-LOX enzymes oxygenate free fatty acid and their large phospholipid esters with identical regio- and stereo-specificity – only possible using “tail-first” entry. This is also supported by the 3D structure of soybean LOX, which lacks a back door [3,4].

Despite the compelling evidence, tail-first entry has been challenged, (or discounted), by others, including in the past decade by Hoffmann, Klinman and coworkers: “our MD simulations provide preference for a “carboxylate-in” binding orientation for LA” in soybean LOX. The remainder of this discussion will thus pertain to the “carboxylate-in” binding orientation.” [23]. So much for MD simulations, in contrast to experimental results. Of course, the “carboxylate-in” orientation is predicted as one of the several orientations for soybean LOX-1 reactions at pH 6 – pH 7 that produce mixtures of *cis-trans* and *trans-trans* conjugated hydroperoxides including a proportion of 9*S*-HPODE [61], but certainly the canonical reaction at high pH producing almost pure 13*S*-HPODE involves only tail-first binding of ionized linoleate [61]. It is pertinent to emphasize here that the said conclusions do not rest on consideration of one lipoxygenase with one substrate forming one product, viz. linoleic acid reacting with soybean LOX-1 to form 13*S*-HPODE. It is based on the experimental evidence stemming from analysis of the broad spectrum of LOX catalysis (positional and stereo specificities and the associated hydrogen abstractions) and the substrate orientation concepts that the experimental evidence dictates [7,9,38,60, 62–64].

### Oxygenation of large esters and its significance -

The rabbit reticulocyte 15-LOX (ALOX-15) was first identified as an enzyme that oxygenates fatty acids esterified in membranes [65,66], analyzed in detail and shown to form 13*S*-hydroperoxy and 15*S*-hydroperoxy products from phospholipid esters of LA and AA respectively [67–69]. In 1985 Jung and coworkers reported on the LOX metabolism of arachidonate esterified in phosphatidylcholine (PC) using multiple sources of lipoxygenase [70]. A cell-free preparation of human neutrophil 5-LOX and 15-LOX formed specifically 15-HETE in the PC, and significantly, no 5-HETE. 15-HETE was also formed in the PC by soybean LOX and rabbit reticulocyte 15-LOX [70]. The stereo-specificity of this type of transformation was demonstrated for soybean LOX-1, with linoleate in a PC ester being converted to the 13*S*-hydroperoxide [71]. Later it was established, and perhaps of physiological relevance, that lyso-PC is an excellent LOX substrate for 15-LOX enzymes [72,73]. These and later studies indicate that substrates for 15-LOX metabolism must enter the enzyme active site tail-first. This line of evidence also holds for 12*S*-LOX [74]. It follows also from this type of experiment, and supported by other indications (and assuming use of the same substrate access channel), that mammalian 5*S*-LOX and plant 9*S*-LOX are prominent enzymes that bind substrate in reverse orientation, with carboxyl end-first entry to the active site.

### A physiologically important enzyme with both modes of substrate entry -

**Fig. 7.**
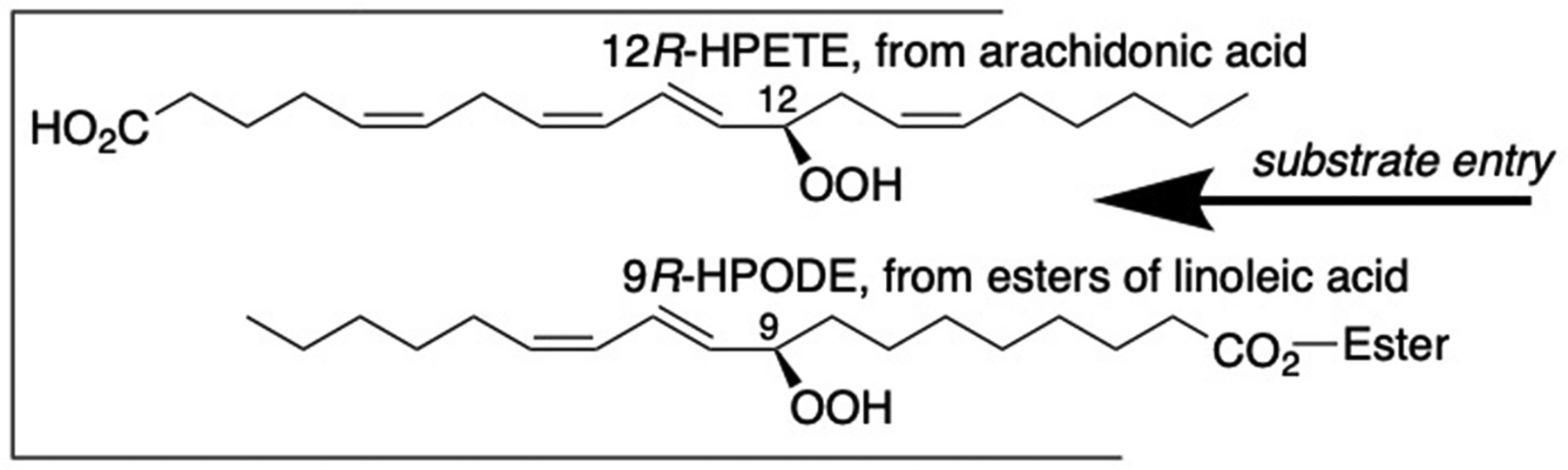
Reverse substrate orientations in the transformations of arachidonic acid or linoleate esters by 12*R*-LOX.

Human 12*R*-LOX was identified and is named from its formation of 12*R*–H(P)ETE, a major skin oxidized lipid associated with psoriasis [75–78]. Notably, the “head-first” orientation with arachidonic acid in forming 12*R*-HPETE is changed to tail-first in the case of linoleate and its ceramide esters, giving 9*R*-hydroperoxides as product, Fig. 7. This orientation rationalizes the 9*R*-LOX metabolism of linoleate esterified in acylceramides in formation of the skin permeability barrier [55, 79,80].

### A modification of the one-way entry concept -

**Fig. 8.**
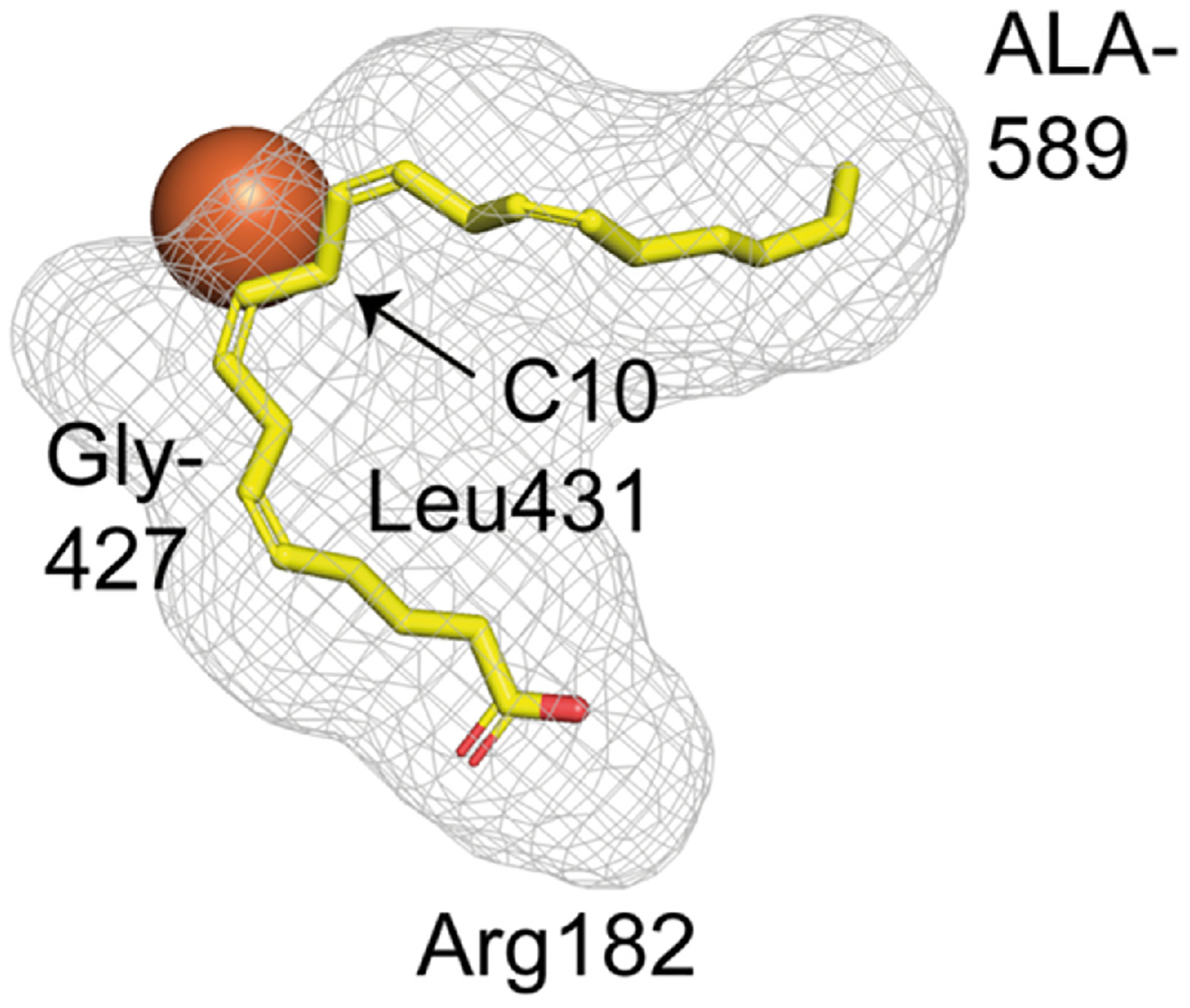
Arachidonic acid bound in the U-shaped substrate channel of 8*R*-LOX (and role of four amino acids) In the crystal structure of *P. homomalla* 8*R*-LOX [24], AA occupies the U-shaped channel (grey mesh) with C10 (arrow) positioned 4.1 Å from the catalytic Fe and “clamped” in this position by Leu431. This channel is open only for tail-first access, the small residue Ala589 allowing AA to slide in further than in 12*S*- and 15*S*-LOX (cf. [83]), leaving the AA carboxyl close to Arg182. Gly427 permits access of O_2_ at C8 via an oxygen channel (see Fig. 8). The substrate channel was outlined with a PyMOL plug-in (CavitOmiX; radius probe 1.5 Å, radius factor 0.83).

In terms of the principles of oxygenation and the relationship to substrate binding orientation, the evidence in support of this model is overwhelming and will not be further reiterated here [7,9,38,62–64]. Nonetheless, there is a potential alternative to carboxyl end-first entry for enzymes such as plant 9*S*-LOX. This entails a putative U-shaped substrate access channel with “tail-first” access at both ends, allowing 13*S*-metabolism through a conventional “tail-first” entry at one end, and at the other end a “backdoor” access to the fatty acid tail for 9*S*-metabolism. The 15*R*-LOX of the bacterium *S. Cellulosum* [81] is another candidate to consider for “back door entry” as otherwise the substrate binding channel and active site would have to accommodate the carboxyl group and at least 15 carbons of the arachidonate molecule. The putative channel is similar in principle to one described for the X-ray crystal structure of *P. homomalla* 8*R*-LOX [82], and which was later defined in the presence of bound arachidonic acid substrate [24], Fig. 8. A difference is a U-shaped channel open at both ends.

The concept of a substrate channel open at both ends was suggested as an explanation for the 9*S*-LOX metabolism of esters of glycerol and lyso-PC [84]. Maize 9*S*-LOX converts 1-linolenoyl-*rac*-glycerol ester mainly to the 9*S*-hydroperoxide [84,85], and in the absence of enzyme-substrate structures it is unclear whether conventional “carboxyl-end entry” is a viable explanation (is there room for entry of the glycerol head group?). More challenging to convention is the A562G mutant producing a modest enantiomeric excess of 9*S*-hydroperoxide from 1-linoleoyl-lyso-PC (61:39 ratio of 9S:9R), indicating some chiral interaction with the enzyme [84]. Lyso-PC is a poor substrate and among the products were prominent hydroperoxides with *trans-trans* conjugated dienes, a sure sign of lack of enzyme control; nonetheless, how to account for the enantiomeric excess of 9*S*-hydroperoxide with lyso-PC esterified on the 1-carbon? The “backdoor” concept is an intriguing proposal, which, pointedly, still follows the well-established (and indisputable!) concept of regular or reverse orientation of substrate.

## 5. Primary Kinetic Isotope Effects (KIE) and Hydrogen Tunnelling

The mass ratios of hydrogen and its two isotopes are 1:2:3, and as hydrogen abstraction is rate limiting in LOX catalysis and some other enzymes, the stronger C-D and C-T bonds will reduce ^D^k_cat_ and ^T^k_cat_ in comparison with ^H^k_cat_. Although theoretical calculations based on bond strength predict KIE ratios of ^H^k_cat_/^D^k_cat_ = 6.9 and ^H^k_cat_/^H^T_cat_ = 16, the measured ratios in LOX reactions are substantially higher and for most enzymes with H-abstraction as rate-limiting usually in the range of 15–20 for deuterium-labeling and 50–75 for tritium. The higher KIEs are attributed to hydrogen (^1^H) tunnelling through the energy barrier rather than over it, and as a consequence of ^1^H taking the lower energy route, the KIE ratios for D/H and T/H are higher than otherwise expected. Tunnelling of small particles is possible on account of the wave-particle duality of quantum mechanics, in this case the wave-like properties of ^1^H [86]. The abstraction of hydrogen occurs not by removal of an H· radical per se, rather by a proton-coupled electron transfer, PCET, (H^+^ + e^−^) [87–90].

Klinman and co-workers have described the mechanism of hydrogen tunnelling of soybean LOX-1 in detail [91–96]. The D-KIE ranged between 60 and 80 and was almost temperature-independent [23,25, 97–100], which suggests a constant hydrogen donor-acceptor distance [23]. The measured D-KIE can vary for different LOX and with experimental conditions and with [11-^2^H_2_]- or [11*S*–^2^H]-linoleate as substrate it ranges between 15 and 80 [98–100]. D-KIE values of 40–80 obviously correspond to ^D^k_cat_ of only 2.5–1.25% of ^H^k_cat_. On a technical level the precision of D-KIE values can be uncertain due to the low conversion rate of the deuterated fatty acids, the extended time lag of some LOX at low temperatures, and errors due to dividing a large and certain number (^H^k_cat_) with a much smaller and relatively uncertain one (^D^k_cat_).

The D-KIE of MnLOX has also been investigated. 13*R*-MnLOX oxidized [11*S*–^2^H]18:*n*-6 with a D-KIE of ca. 38 at +8°C to ca. 20 at 50°C with a 7-fold increase in the reaction rate [99]. The temperature-dependent D-KIE of 13*R*-MnLOX may suggest a combination of hydrogen tunnelling and semi-classical transitional state at high temperatures [99]. D-KIE estimates of 9*S*-MnLOX varied between ca. 40 (21°C) and 60–80 (10–30°C) with [11,11-^2^H_2_]LA as substrate [99,100]. Although the D-KIE was slightly temperature-dependent, the results “strongly support a non-classical, full tunnelling mechanism” [100].

It might be added that secondary isotope effects on the D-KIE are small but might increase the primary D-KIE [25,98,101]. This can be illustrated by 11*R*-LOX (*F. oxysporum*), which oxidized [11*S*–^2^H]18:2*n*-6 by abstraction of the 11*R* hydrogen at half the rate of unlabeled LA (D-KIE 2) [102]. Although the *pro-S* deuterium at C-11 does not directly participate in the reaction, it nevertheless stabilizes the C–H bond and reduces the rate of *pro-R* hydrogen abstraction, (ref. [103] for review).

## 6. Oxygen Access and O_2_ Channels

In contrast to monooxygenases such as cytochrome P450s that bind and hold oxygen for catalysis [104,105], for LOX enzymes all the evidence supports O_2_ having free reign to wherever diffusion takes it (e.g. Ref. [93]). So, to achieve a regiospecific and stereospecific outcome the molecule of O_2_ has to come face-to-face with the appropriate reactive carbon radical - while avoiding other potential reactive positions on the fatty acid pentadiene. Although in principle the carbon radical could be localized by a twisting of the otherwise planar pentadiene, thus ensuring a single product outcome [106], more likely a combination of two circumstances controls the oxygenation: the protein structure shields other sites from encountering the O_2_ molecule and/or the O_2_ is directed to a position or pocket in the protein face-to-face with the appropriate site on the fatty acid chain. How it gets there is the subject of many a computational and experimental analysis and has not been unambiguously answered.

O_2_ might reach the metal center via the substrate access channel or via gas migration channels through the protein. Taking the same pathway in as the substrate seems obvious, but apparently most computational analyses favor diffusion pathways through the protein structure. A route through the protein was investigated in soybean LOX-1 by Klinman and coworkers by a PyMOL plugin (Caver) for detection of dynamic protein pathways and by extensive kinetic investigations [21, 107–109]. In summary, a route named channel A was “identified as the preferred route of O_2_ delivery to the active site of catalysis” and lined with Ile553. The Ile553Trp replacement diminished O_2_ availability at the active site and also altered the product profile, which was attributed to restriction of channel A. Another interpretation remains possible until the structures of the soybean LOX-1:AA complex and the Ile553Trp mutant are solved.

**Fig. 9.**
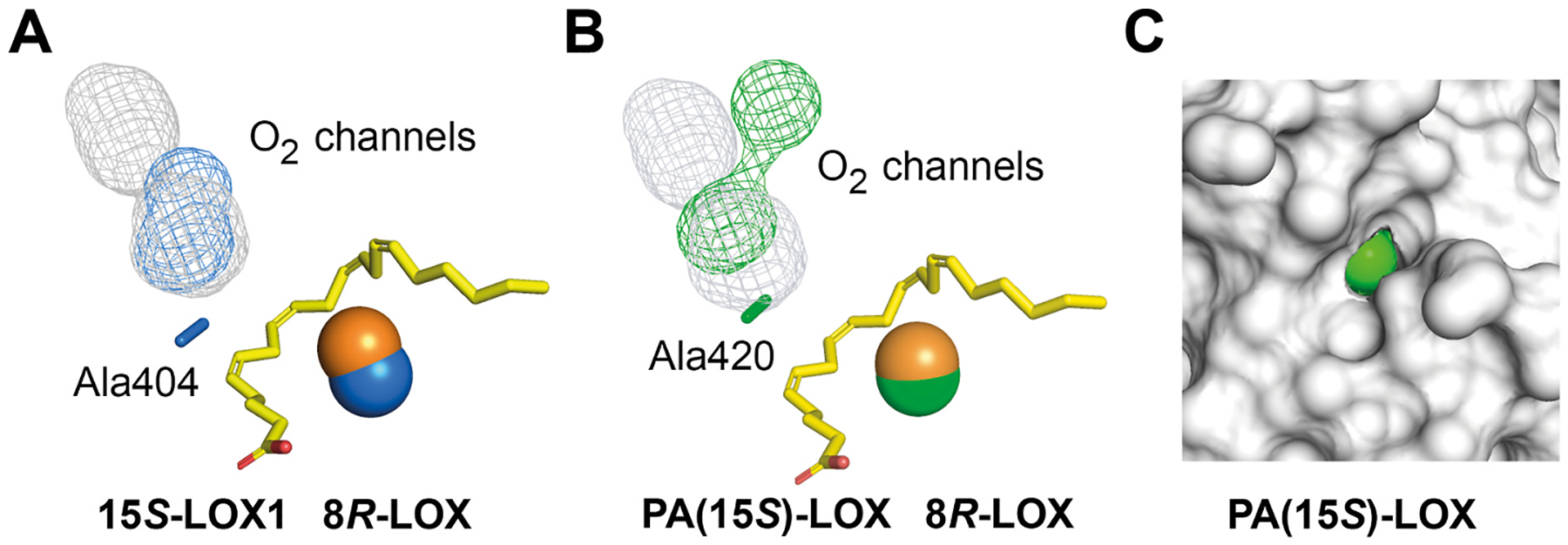
Predicted oxygen channels of 8*R*-LOX, 15*S*-LOX1, and PA(15*S*)LOX **A**. The presumed oxygen channel of 15*S*-LOX1 is marked in blue (mesh) with Ala404 and the catalytic Fe in blue and placed in superposition with the oxygen channel of 8*R*-LOX in grey (mesh), the catalytic Fe in orange, and AA in the active site in yellow (cf. Fig. 7). B. The presumed oxygen channel of *Pseudomonas aeruginosa* PA-LOX is marked in blue (mesh) with Ala420 and Fe in blue and in superposition with 8*R*-LOX as in A. C. Part of the protein surface of PA-LOX in grey with the small orifice of the oxygen channel at the surface in green. The oxygen channels were estimated with a PyMOL plug-in (CavitOmiX; radius probe 1.5 Å, radius factor 0.83).

O_2_ channels are also proposed, for example, in 15*S*-LOX1, 8*R*-LOX, PA-LOX, and CspLOX2 [24,110–113]. These tunnels connect the protein surface with residues of the Gly/Ala switch and the metal center, Fig. 9. Structural analysis of PA-LOX, its Ala420Gly mutant, and analysis of the product profile provided convincing support for an O_2_ access route [111].

## 7. Enzyme Residues That Control Regio- and Stereo-specificity

Investigations on the role of specific amino acid residues in controlling fatty acid oxygenation within the substrate access channel are well reviewed in the literature [7,62,60,114–116]. They will be considered here only in overview. Structural insights were first demonstrated as rational changes to product formation by mutagenesis of amino acids deep in the substrate binding pocket of 12-LOX and 15-LOX-1 [83]. Subsequently the understanding was amplified [117], and extended to three structurally significant residues in control of 15-LOX-1 catalysis [64,116]; the triad concept partly applied to 12-LOX although is not applicable to 15-LOX-2 and 12*R*-LOX [64,115,118]. Analysis of a series of chimeric enzymes located two residues largely responsible for the substrate orientation and 8*S* or 15*S* product specificity of murine 8*S*-LOX and human 15-LOX-2 [119]. As expected, considerable attention is directed towards the reaction specificity of soybean LOX-1 (e.g. Refs. [21,120–122].

**Fig. 10.**
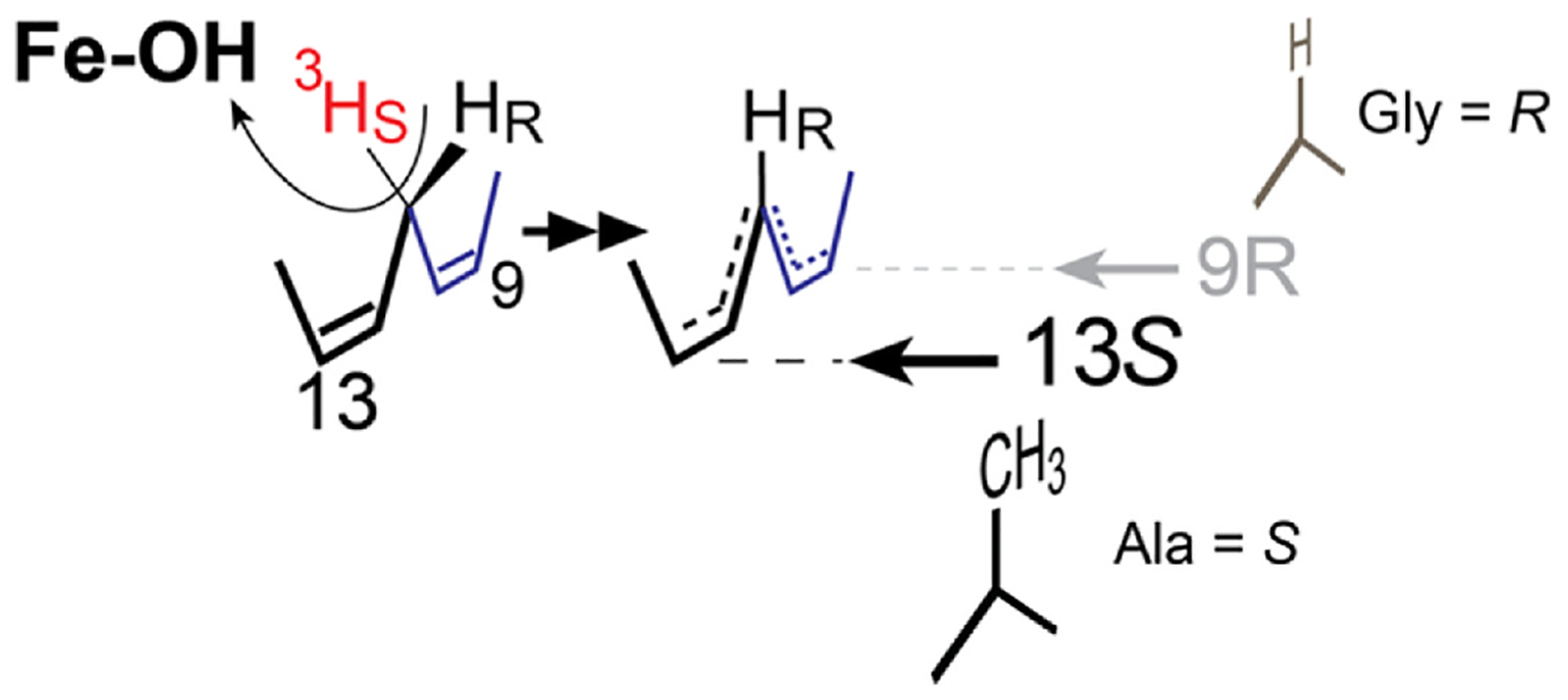
Linoleic acid is oxygenated with antarafacial H-abstraction/oxygenation and 13*S*/9*R* stereochemistry by 542 Ala/Gly substitution in soybean LOX-1 Metabolism of stereospecifically labeled [11-3H]linoleic acids was used to show these relationships [121].

**Fig. 11.**
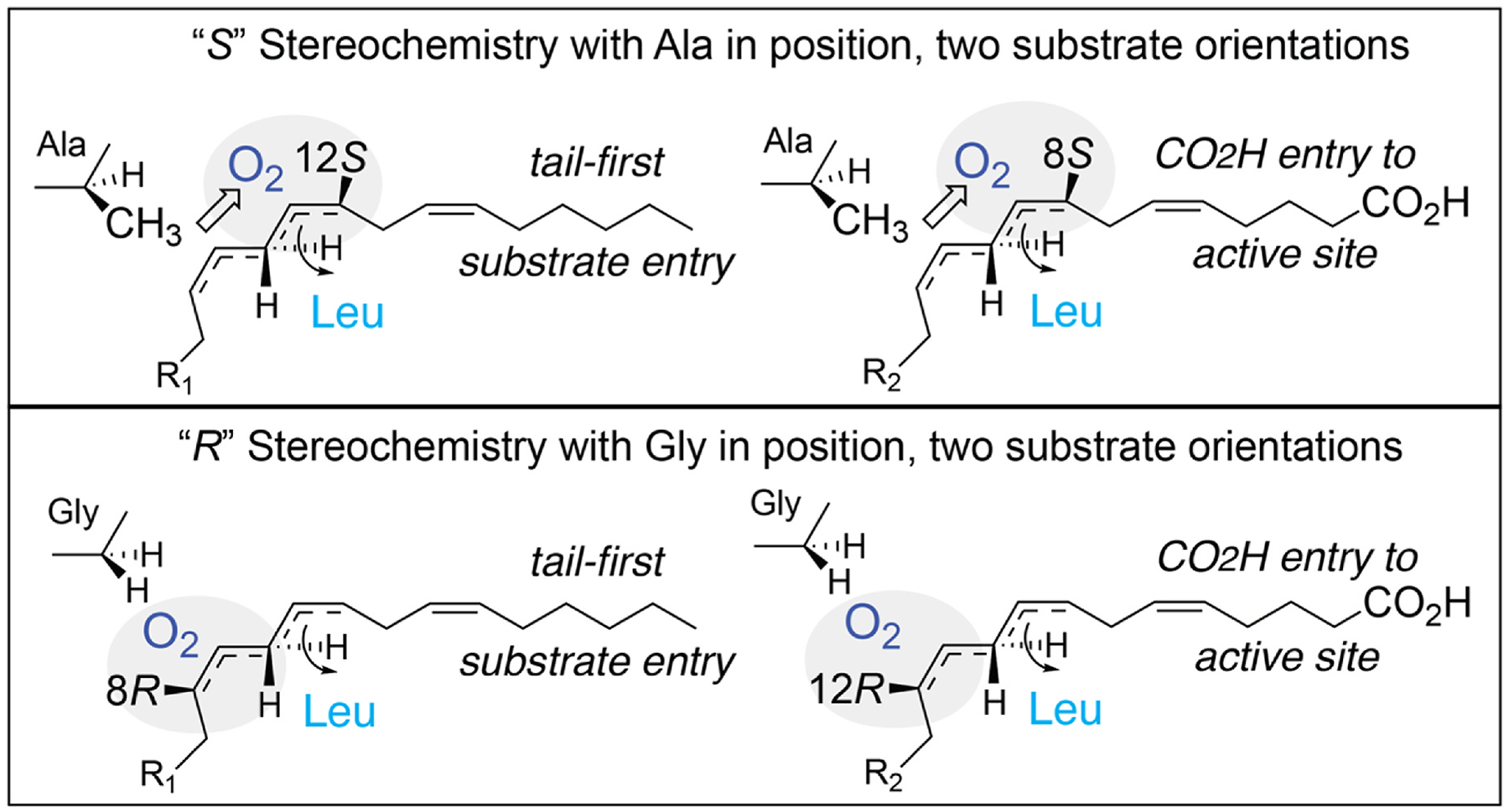
Comprehensive model of the principle of *R* or *S* stereo control in lipoxygenases with alternative substrate orientations and Ala-Gly switch. In these examples, formation of four different products is represented in lipoxygenase active sites of related structure. Top: *S*-stereochemistry products are formed when Ala (or occasionally an equivalent residue), in cooperation with an invariant Leucine (illustrated in Ref. [24]), diverts O_2_ towards 8*R* or 12*S*, depending on orientation of the fatty acid substrate. Below: *R*-stereochemistry product formation, 8*R* or 12*R*, with Gly in the critical position, again dependent on substrate orientation and influence of the invariant Leucine in the LOX active site.

In the development of these concepts, further insights came from identification and characterization of LOX enzymes with “*R*” stereo-specificity [78,123,124]. Amusingly, considering the prevalence of “alanine scanning” for removing roles of residues in proteins and enzymes, an Ala or Gly substitution has a general role in control of LOX stereospecificity: an Ala in the critical position next to the bound substrate gives *S* stereochemistry products and Gly confers *R* stereospecificity [63,124]. The Ala-Gly switch influences the access of O_2_ to one end or other of the activated pentadienyl radical, thus producing *S* or *R* stereochemistry, Fig. 10 [121]. There are a few exceptions [125–127], but most significantly the Ala/Gly switch illustrates the principle, or at least a part of the principle of R/S stereocontrol. Placement of an invariant Leu (8*R*-LOX Leu-431), one turn of the helix from the Gly/Ala switch, acts as a shield and is coupled to the switch allowing O_2_ access, in the example in Fig. 11, to the C8 or C12 ends of the pentadiene.

It turns out that, for example, 12*S*-LOX and 12*R*-LOX are not closely related from a mechanistic point of view. The close “partner” of 12*S*-LOX is 8*R*-LOX (or 12*R*- and 8*S*-LOX) as each pair catalyzes reaction with O_2_ on the same face of the reacting substate and at one end or the other of the activated pentadiene [124]. Each pair also share the same antarafacial hydrogen abstraction, demonstrated using stereospecifically labeled [11-^3^H]linoleic acid for an Ala-Gly mutant of soybean LOX-1, in this case giving linoleate 9*R* and 13*S* hydroperoxide products, Fig. 10 [121]. Taking these findings together allowed construction of a comprehensive scheme to model the reactions of LOX enzymes for any designated regiospecificity and with *R* or *S* stereochemistry, Fig. 11 [63].

## 8. Last Step: Formation of Hydroperoxide with Regeneration of the Fe^3þ^ Enzyme

It is apparent that the ROO· to ROOH step is well-controlled by the enzyme because it happens fast and is not associated with the facile side reactions of a free peroxyl radical. Consider comparative time-scales. A free peroxyl radical (ROO·) has an estimated half-life of 7 s [128], and when surrounded by double bonds, as in a “mid-chain” arachidonate peroxyl radical, a preferred product outcome is addition to the nearby *cis* double bond with formation of endoperoxides [129]. As none of these appear in LOX-controlled biochemistry it is apparent that the free ROO· radical is quenched before it can escape the active site. To give some idea of the time frame of the final step of hydroperoxide formation in LOX catalysis, consider that the LOX enzymes with high turnover numbers (*kcat* ~ 300/s e.g. Refs. [92,130,131]) have the entire catalytic cycle from acquisition of substrate to release of product over in ~3 ms, necessitating a substantially shorter timeframe for the LOX-controlled ROO· → ROOH transformation. Even the slowly reacting LOX enzymes (*kcat* ~1/s) must execute the last step promptly, for it is earlier step(s) in the cycle that are rate-limiting and again there is formation and release of a clean hydroperoxide product, e.g. Refs. [132–134].

**Fig. 12.**
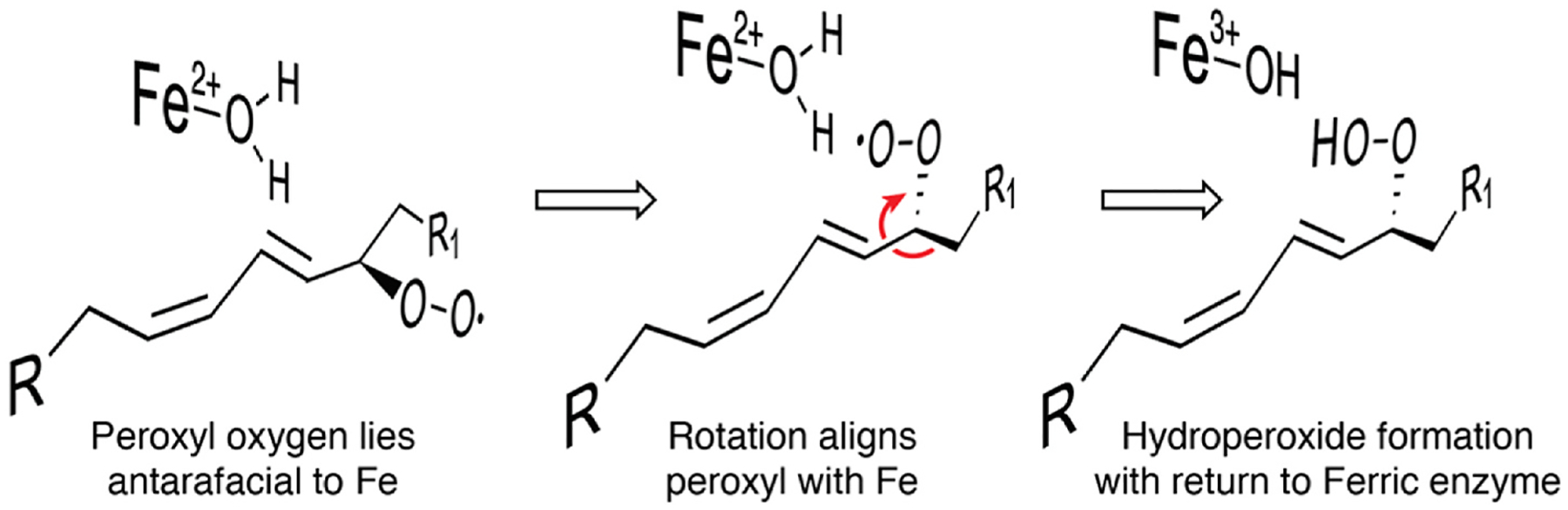
Perspective view on completion of the catalytic cycle with reduction of the fatty acid peroxyl radical Left: Reaction with O_2_ leaves the fatty acid peroxyl radical lying antarafacial to the iron center. Middle: Rotation of the alpha carbon chain brings the peroxyl to a suprafacial position, permitting PCET transfer of proton and electron with reduction to the final hydroperoxide product and oxidation of the metal center back to the Fe^3+^ active enzyme (right side). This drawing is inspired by figures with computed distances and detailed analyses of PCET in 8*R*-LOX [135].

This ROO· to ROOH transformation is hard to approach experimentally and has received minimal attention, especially compared to the initial hydrogen abstraction. Nonetheless, a comprehensive computational analysis of 8*R*-LOX does an excellent job of reviewing the issues and formatting a model for this last step [135]. Initially the fatty acid peroxyl lies antarafacial to the metal center and has to rotate into a suprafacial position, Fig. 12. Although one might imagine that steric interactions between the substrate and the active site could inhibit this rotation, perhaps surprisingly, computational analysis indicated the impact of the active site was not that significant [135]. Rotation of the carbon chain at 8,9 will bring the peroxyl suprafacial, then computations favor PCET transfer of an iron-liganded proton to the oxygen as an electron leaves the iron center, producing the final fatty acid hydroperoxide and regenerating the active Fe^3+^ enzyme [135].

As Mn-LOX catalyze suprafacial H-abstraction and oxygenation [102,20], they do not have this rotation requirement in completing the catalytic cycle. Nonetheless, upon a single Phe337 to Ile mutation that switches MnLOX to antarafacial reaction, the substrate rotation with HPODE formation is accomplished [136]. This observation is consistent with the deduction that no enzyme residues dedicated to the final PCET transfer (MnLOX would not need them) and it is rotation of the substrate that allows completion of the catalytic cycle.

Oh! And one final step in catalysis, release of the hydroperoxide product and its exit from the LOX protein. In some enzymes, exit of the product can be rate-limiting (e.g. Ref. [137]), and release is a measurable parameter in LOX catalysis, at least for soybean LOX-1 [92,138], which exhibits a high pKa (~ pH 8) attributable to substrate binding and a second lower pKa (~pH 7) influencing product release [92]. Within this picture in soybean LOX-1 and other lipoxygenase, much attention is given to the role of an Arg or other basic residue near the entrance to the substrate-binding channel (e.g. Refs. [24,139–141]). This residue has a proposed role in binding and positioning the substrate for catalysis, although what might be its effect on product release? Not examined or explained, it so appears.

## 9. Concluding Remarks

One of the objectives in writing this review is to highlight areas of interest that remain open to further mechanistic insights. Hopefully the reader will appreciate the current incomplete understanding of activation of the metal ligand for LOX catalysis. Can this be approached and further clarified with the classic models or new LOX enzymes? One can also surmise that substrate entry and product exit is unclear (as is true for LOX and many other enzymes). The developing capabilities of AF2 and beyond will contribute here.

Finally, current technologies place limits, and at the present time there are minimal capabilities in equating primary or 3D structure with catalytic outcomes in LOX biochemistry, highlighting major research possibilities for the future.

## Footnotes

1 Of historical and mechanistic interest, adding a molar excess of 13-HPODE to milligrams of soybean LOX creates a purple-colored protein, a ferric-13-HPOD complex, long considered as a potential participant in the normal catalytic cycling (e.g. Refs. [40,142–144]), although discounted in favor of the ferric (yellow) enzyme in later kinetic and computational analyses [107,135].

## Abbreviations:

AA: arachidonic acid
AF2: AlphaFold-2
eLOX3: epidermal lipoxygenase-3
H(P)ETE: hydro(pero)xy-eicosatetraenoic acid
H(P)ODE: hydro(pero) xy-octadecadienoic acid
KIE: kinetic isotope effect
LA: linoleic acid
LOX: lipoxygenase
PC: phosphatidylcholine
PCET: proton-coupled electron transport
RMSD: root mean square deviation
SOD: superoxide dismutase
